# Insufficient stem antetorsion and lower cup abduction is a combined risk factor for posterior hip dislocation in patients undergoing THA for femoral neck fractures: a retrospective analysis

**DOI:** 10.1186/s12891-024-07199-2

**Published:** 2024-01-30

**Authors:** Zhuokai Li, Yang Yang, Shengyang Guo, Ju Liu, Xiaoxiao Zhou, Houlin Ji

**Affiliations:** 1 Department of Orthopedics, Shanghai University of Medicine & Health Sciences Affiliated Zhoupu Hospital, Shanghai, China; 2 Department of Orthopedics, Taizhou Hospital of Zhejiang Province Affiliated to Wenzhou Medical College, Zhejiang, China; 3 Jinji Lake Community Health Service Center of Suzhou Industrial Park, Suzhou, China

**Keywords:** Primary total hip arthroplasty, Dislocation, Abduction, Femoral neck fracture, Cementless stem, Stem antetorsion

## Abstract

**Background:**

The role of acetabular and femoral component positions with respect to the risk of post-operative instability and dislocation remains debated. In this study, we aimed to identify potential risk factors for early dislocation following primary total hip arthroplasty (THA) for displaced intracapsular femoral neck fractures (FNF) using radiological measurements.

**Methods:**

We retrospectively analyzed data for patients who underwent cementless primary THA for FNF using a posterolateral approach between January 2018 and December 2021. Follow-up duration, age, sex, affected side, and mean time from THA to dislocation were recorded. Leg-length inequality, abductor lever arm, vertical and horizontal femoral offsets, vertical and horizontal hip centers of rotation, abduction, anteversion of the acetabulum and femoral prosthesis, and combined anteversion were measured.

**Results:**

The study sample included 17 men and 34 women, with 21 and 30 patients undergoing left- and right-hip operations, respectively. The mean patient age was 70.18 ± 7.64 years, and the mean follow-up duration was 27.73 ± 13.52 months. The mean time between THA and dislocation was 1.58 ± 0.79 months. Seven patients (13.73%) sustained posterior dislocation of the hip. The abduction angle (36.05 ± 6.82° vs. 45.68 ± 8.78°) (*p* = 0.008) and anteversion of the femoral prosthesis (8.26 ± 4.47° vs. 19.47 ± 9.01°) (*p* = 0.002) were significantly lower in the dislocation group than in the control group. There were no significant differences in other parameters.

**Conclusions:**

Insufficient stem antetorsion combined with lower abduction angle of the acetabular component were associated with a high risk of dislocation, especially in patients with deep flexion or internal rotation of the flexed hip joint and knees, or in patients with a stiff spine or anterior pelvic tilt, impingement may then occur in the neck of the prosthesis and cup component, ultimately resulting in posterior dislocation. These findings could remind surgeons to avoid simultaneous occurrence of both in THA surgery. These results provide new insight into risk factors for hip dislocation in patients undergoing primary THA for FNF and may aid in reducing the risk of instability and dislocation.

**Level of evidence:**

Prospective comparative study Level II.

## Background

Total hip arthroplasty (THA) is one of the most frequently performed orthopedic procedures and was declared the most successful in the twentieth century [1]. Dislocation is a leading early complication of THA, with dislocation rates after primary THA currently ranging from 1.5 to 2%. The risk of dislocation after primary THA for osteoarthritis has been reported as 0.3–10% [2], with the rate of dislocation after revision THA for instability rising up to 15–20% [3]. In the USA, instability/dislocation is the most common indication for revision THA, accounting for 22.5% of revisions [1]. After the first dislocation, 60% of patients sustain recurrent instability, and 50% require revision surgery [4]. A dislocated THA results in tripling of hospital costs compared with an uncomplicated THA [5]. Both patient- and surgery-related factors such as age, body mass index, American Society of Anesthesiology score, alcohol intake [6], surgical approaches [7], femoral neck fractures (FNFs) [8],femoral neck length, femoral head size [9], acetabular cup positioning [10], hip offset, and leg length restoration [11, 12] have been shown to increase the risk of dislocation after THA.

The role of acetabular component orientation in dislocation formation has been extensively investigated. A recent systematic review of cup positioning in primary THA identified that most studies did not report a statistically significant reduction in the incidence of dislocation for cups placed within the Lewinnek safe zone [13], and the role of the acetabular and femoral component positions with respect to the risk of post-operative dislocation is still debated.

Prior studies have indicated that THA performed in elderly patients for the treatment of acute FNF is associated with a higher rate of dislocation [8]; however, the risk of dislocation after primary THA for FNF has not been fully elucidated. Thus, we aimed to identify the potential risk factors for early hip dislocation following primary THA for FNF using a standard posterolateral approach.

## Materials and methods

### Patients

We enrolled 55 consecutive patients with displaced intracapsular FNF who underwent primary THA at our institution between January 2018 and December 2021. Patient selection was guided by a set of inclusion and exclusion criteria, with the inclusion criterion being patients requiring non-cemented primary THA for FNF. Patients were excluded if they had undergone revision THA for an indication other than primary THA, or if they received a cemented femoral stem during their index arthroplasty. Patients with substantial neurological or musculoskeletal disorders that would adversely affect gait or early weight-bearing after surgery and those with missing radiological or clinical data were also excluded. Two patients with postoperative dislocation were excluded because their primary THA was performed at another hospital, and one patient was excluded for revision THA. This study was approved by by the Institutional Ethics Committee of Zhoupu Hospital, Affiliated to Shanghai University of Medicine & Health Sciences. Written informed consent was waived by the Institutional Ethics Committee of Zhoupu Hospital, Affiliated to Shanghai University of Medicine & Health Sciences.

### Surgical procedure

All surgeries were performed using the standard posterolateral approach by a senior surgeon with more than 30 years of experience. To improve exposure, the proximal iliopsoas muscle was partially released from the lesser trochanter. All patients received uncemented acetabular and femoral components, most of the socket was fixed with two auxiliary screws to augment cup anchoring, and short external rotators were repaired with absorbable sutures using a transosseous technique. Preoperative templating was not routinely performed in any of the patients. If the patient’s physical condition permits, the patient should be allowed to walk with a walking aid for three days after surgery.

### Recorded demographics and radiological parameters

**Fig. 1:**
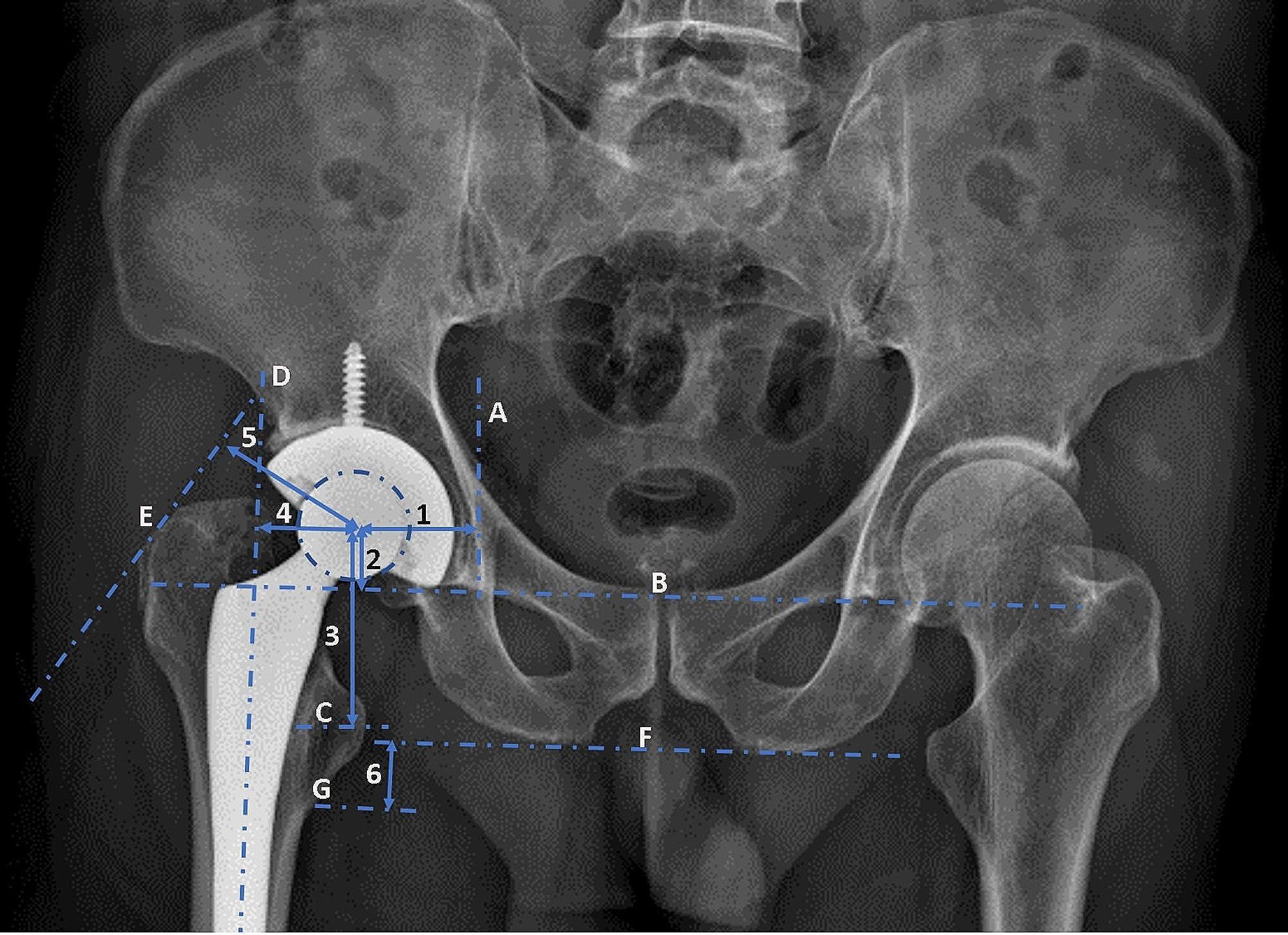
Schematic diagram showing the measurement of biomechanical parameters determined by postoperative radiographs after total hip arthroplasty. 1, horizontal hip center of rotation; 2, vertical hip center of rotation; 3, vertical femoral offset; 4, horizontal femoral offset; 5, abductor lever arm; 6, limb length. (**A**), vertical teardrop line; (**B**), horizontal tear drop line; (**C**), midline lesser trochanter; D, femoral shaft axis; E, tangential line to the greater trochanter; F, bi-ischial line; G, lower line of lesser trochanter

**Fig. 2:**
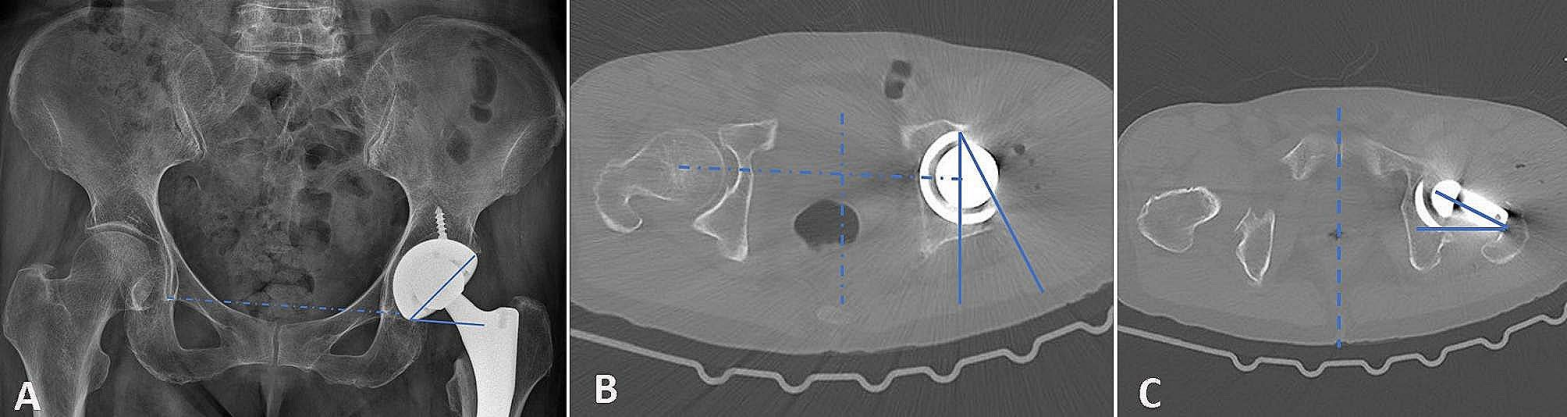
Schematic diagram showing the measurement of the component parameters. (**A**), the abduction angle of the cup was defined as the angle formed by the parallel line of the connecting the two teardrops and the line connecting the upper and lower ends of the open plane of the cup on an anteroposterior radiograph of the pelvis. (**B**), the anteversion angles of the cup defined as the angle between a line connecting the lateral anterior and posterior margins of the acetabular component and the sagittal plane defined as the plane perpendicular to a line connecting two identical points on either side of the pelvis on an axial plane of 2-dimensional computed tomography. (**C**), the anteversion angles of the femoral component were defined as the angle between a line of the head-neck axis and the coronal plane defined as the plane perpendicular to a line connecting two identical points on either side of the pelvis on an axial plane of 2-dimensional computed tomography

Patient demographic data, including age, sex, stem design, mean time from THA to dislocation (months), and follow-up time (months), were recorded. For all postoperative measurements, we used the earliest postoperative images available prior to dislocation. Postoperative measurements included the vertical and horizontal femoral offsets, abductor lever arm, and horizontal and vertical hip centers of rotation, according to a previous study (Fig. 1) [14]. Leg length inequality was measured as described in a previous study (Fig. 1) [15]. If the leg length in the operated hip was longer than that in the non-operated hip, ‘‘+’’ was recorded and vice versa. The cup abduction angles were measured on anteroposterior (AP) radiographs of the pelvis (Fig. 2A) [16]. The anteversion angles of the cup were measured using axial computed tomography (CT) scans (Fig. 2B) [17], whereas the anteversion angles of the femoral component were measured using axial CT scans (Fig. 2-C) [18] with a picture archiving and communication system (PACS; GE Healthcare, Chicago, Illinois, USA). During CT scanning, the pelvis and knee joint were maintained in a neutral position in order to keep the femoral condyle line parallel to the CT-table. All the patients were managed using the same comprehensive perioperative pain management and rapid rehabilitation protocol.

### Statistical analysis

Statistical analyses were performed using IBM SPSS software (version 23.0; IBM Corporation, Armonk, NY, USA). Continuous variables are presented as mean ± standard deviation. An independent-sample *t* tests were used to compare two groups. Categorical variables are shown as numbers and were compared using the chi-square test or Fisher’s exact test when the expected count was less than five. The level of significance was set at *p* < 0.05.

## Results

One patient in the dislocation group had − 5.17° anteversion of the acetabulum, 36.25° abduction, and 30.65° combined anteversion. As the cause of posterior dislocation was retrograde acetabular prosthesis, its diagnosis was definitive, and the patient was excluded from the study. Seven patients (13.73%) sustained posterior dislocation of the hip (seven patients/seven hips), including five single dislocations, one double dislocation, and one patient with more than two dislocations.

The study sample included 17 men and 34 women, with 21 and 30 patients undergoing left- and right-hip operations, respectively. The mean patient age was 70.18 ± 7.64 years (range: 49–82 years), and the mean follow-up duration was 27.73 ± 13.52 months (range: 7.47–47.21 months). The mean time between THA and dislocation was 1.58 ± 0.79 months (range: 0.62–2.70 months).

There were no significant differences in age, sex, affected side, follow-up duration, abductor lever arm, vertical/horizontal femoral offsets, vertical/horizontal hip centers of rotation, leg-length inequality, anteversion of the acetabulum, or combined anteversion between the dislocation and control groups (Tables 1 and 2).

**Table 1:** Demographic data of the participants

Basic Characteristics	Dislocation group (*n* = 7) (range)	Control group (*n* = 44) (range)	p-value
Age (years)	67.86 ± 9.89 (55–82)	70.55 ± 7.30 (49–82)	0.393
Sex (male/female)	3/4	9/35	0.194
Left/right	2/5	19/25	0.466
Follow-up duration (months)	29.09 ± 12.42 (7.47–42.77)	27.51 ± 13.81 (8.35–47.21)	0.778

Data are presented as the mean ± standard deviation or number of patients. Differences are considered significant at *p* < 0 0.05

**Table 2:** Characteristics of patients with hip dislocation after total hip arthroplasty and controls

Parameters	Dislocation group (*n* = 7) (range)	Control group (*n* = 44) (range)	p-value
Leg length inequality (mm)	-3.57 ± 8.57 (-16.16–9.07)	-6.59 ± 6.18 (-18.76–9.65)	0.260
Abductor lever arm (mm)	-2.25 ± 6.69 (-12.27–2.73)	-1.80 ± 10.97 (-19.25–51.45)	0.916
Horizontal femoral offset (mm)	-4.80 ± 6.73 (-13.57–3.48)	-9.38 ± 7.77 (-34.94–5.87)	0.148
Vertical femoral offset (mm)	-11.42 ± 7.44 (-20.07–-1.27)	-9.83 ± 6.80 (-26.07–7.25)	0.573
Horizontal hip center of rotation (mm)	6.84 ± 2.34 (2.88–9.51)	4.42 ± 4.97 (-10.46–18.34)	0.241
Vertical hip center of rotation (mm)	-7.17 ± 5.36 (-13.18–-1.15)	-3.21 ± 5.41 (-14.63–7.81)	0.078
Abduction (°)	36.05 ± 6.82 (28.76–46.39)	45.68 ± 8.78 (23.33–62.49)	0.008
Anteversion of acetabulum (°)	28.50 ± 11.41 (9.99–43.53)	24.77 ± 9.90 (5.74–43.07)	0.368
Anteversion of femoral prosthesis (°)	8.26 ± 4.47 (2.91–14.78)	19.47 ± 9.01 (6.23–40.61)	0.002
Combined anteversion (°)	36.76 ± 12.68 (20.09–52.49)	44.24 ± 12.70 (17.31–79.63)	0.154

Data are presented as the mean ± standard deviation or number of patients. Differences are considered significant at *p* < 0.05

The incidence of dislocation was significantly associated with anteversion of the femoral prosthesis. More specifically, the dislocation group exhibited significantly lower anteversion of the femoral prosthesis (8.26 ± 4.47°; range: 2.91–14.78°) when compared with the control group (19.47 ± 9.01°; range: 6.23–40.61°) (*p* = 0.002). Meanwhile, the abduction angle of the cup was significantly lower in the dislocation group (36.05 ± 6.82°; range: 28.76–46.39°) than in the control group (45.68 ± 8.78°; range: 23.33–62.49°) (*p* = 0.008) (Table 2).

## Discussion

Dislocation of the THA is defined as a loss of contact between the femoral head and the acetabular component, which requires intervention to relocate the joint. Many factors contribute to stability and dislocation in patients with THA, including surgical factors such as soft tissue tension, component positioning, and femoral head size [19].

The surgical approach has been recognized as a potential factor influencing THA stability and abductor function [20]. Exposure of the hip and proximal femur requires the division of the posterior hip capsule and external rotators. The main disadvantage is a higher posterior dislocation rate [21, 22]. Despite the improved clinical results observed with posterior soft tissue repair, skepticism remains regarding the long-term integrity of the results [23]. Deficiencies in native tendon-to-bone attachment have been reported in 43% of piriformis repairs and 57% of conjoined tendon repairs [24]. After tendon-to-bone repair, deficient tendon–bone interfaces are initially filled with fibrovascular tissue, following which tissue remodeling and scar formation between the tendon and bone occur [25]. After 3 months, muscle atrophy may lead to a loss of function in those undergoing repair of the short external rotator tendons [24]. However, enhanced posterior capsule and anatomic short external rotator repair have been associated with improved postoperative stability at the 4-year follow-up when the short external rotator and posterior capsule are repaired in separate layers using a transosseous technique and number 2 Ethibond sutures. Notably, patients were mobilized without weight-bearing restrictions on the first postoperative day and were instructed to avoid hip flexion greater than 90° and any internal rotation for 6 weeks after surgery [26]. Another study reported that dislocation rates for the posterior approach can be significantly reduced to as low as 0.7% when anatomical repair of the posterior capsule and external rotators is combined with increased anteversion of the cup component [27]. Thus, we suspect that lack of repair for the posterior capsule, rough short external rotator repair, and limited post-operative rehabilitation may have impaired structural and functional healing of the posterior hip envelope.

Impingement is considered the final common pathway for instability and dislocation [28]. Component orientation and implant choice both directly affect the safe range of motion in THA, with impingement potentially occurring as a result of component malpositioning; suboptimal head diameter, head-neck ratio, or geometry; or socket depth. Impingement has been defined as a mechanical abutment between the metal femoral neck and cup liner, or as bone-to-bone contact, such as that between the greater trochanter and pelvis [29]. Osseous impingement and soft tissue tension can only decrease this range of motion. Therefore, optimum positioning of the components is necessary to avoid a decrease in the stable range of motion owing to prosthetic impingement.

Although component positioning has been shown to play an important role in hip stability and risk of postoperative dislocation, there is currently no consensus regarding the safe zone for acetabular component positioning. These differences among studies may be attributable to differences in the surgical approach, methods of measuring component positioning, and limitations in statistical power [13, 30–32]. Computer modeling studies have indicated that the abduction angle for optimal cup position ranges from 45–55°. Angles < 55° require an anteversion of 10–20° for both the stem and cup to minimize the risks of impingement and dislocation [33]. Studies on the 3-D orientation of the acetabular cup have shown that the majority of dislocations have an acetabular cup position that resides within the “safe zone” [34]. Recent studies have questioned the validity of this so-called “safe zone” in explaining dislocations and the variety of definitions used [35]. These insights also require reevaluation of the traditionally advised orientation of the femoral component. When regarding the “safe zone” for combined anteversion of 25°–50°, a widespread variation in results can be observed in the literatures. The recommendations and “safe zones” used to date fail to predict or explain the majority of dislocations [36]. In this study, the control group had a combined anteversion angle > 44°, while the posterior dislocated THA was within the “safe zone”. It cannot be concluded that THA with low or high combined anteversion is prone to dislocation.

In contrast to that of the femoral component, the influence of acetabular component orientation on the risk of dislocation has been extensively investigated. Traditionally, a “safe zone” between 10° and 15° anteversion of the neck of the femoral stem in the transverse plane has been used as a guideline for placement of the femoral component [37]. Other studies reported substantially higher anteversion values. One study described wide variations in femoral anteversion in the standing position. Before surgery, more than 80% of patients had values outside of the “safe zone”—a rate that increased to 85% after THA [38]. In another study, similar variations in native femoral anteversion were observed (–15º to 30°) [39]. Whether femoral component anteversion affects hip joint stability remains controversial. One study claimed that there is no evidence on the optimal femoral component anteversion or for the “safe zone” of 10° to 15° [36], although some studies have demonstrated that low femoral anteversion is associated with a higher risk of posterior dislocation and that patients with high femoral anteversion were at risk for anterior dislocation [40, 41].

According to our results, the horizontal and vertical hip centers of rotation of the acetabular component remained unchanged after the procedure (*p* = 0.148 and 0.573, respectively) without inward or upward shifts of the socket, which would not decrease the offset of the hip or substantially increase the risk of bone-on-bone impingement [42]. In our cohort, the dislocation rate was 13.73%. However, a significantly decreased abduction angle and insufficient stem antetorsion was observed in patients with dislocation vs. controls. Our results suggest that the influence of femoral component anteversion on hip stability has been underestimated. We hypothesize that patients with certain pelvic dynamics are at a higher risk of THA instability. Other authors have suggested that patients may develop instability despite optimal component orientation because they have abnormal spinopelvic dynamics characterized by restricted pelvic tilt from the standing to sitting position [43], or in patients with a stiff spine or anterior pelvic tilt [44, 45]. In our cohort, dislocation usually occurred when patients tried to pick up an object from the ground with deep flexion or internal rotation of the flexed hip joint and knees. Likewise, noncompliance is more prevalent in these patient populations, and such actions are not strictly avoided [46]. During these movements, a lower abduction angle of the cup combined with an insufficient stem antetorsion, both of which may have shifted the neck of the prosthesis posterosuperiorly, increasing the workload on the short external rotator. In such cases, inadequate tissue tension and joint hyperlaxity cannot stabilize the femoral head in the acetabulum. This may result in increased contact between the neck of the prosthesis and the superior margin of the cup component, leading to primary impingement and posterior dislocation of the femoral head.

This study had some limitations, including its small sample size and retrospective design. The monocentric nature of the data and lack of randomization may have also resulted in selection bias. Dislocation after THA is a multifactorial problem, and this study addressed only parts of this issue. Some parameters were measured on plain AP radiographs and 2-dimensional computed tomography, the accuracy of which is inevitably affected by radiological positioning and interobserver variability. Further, we did not evaluate the integrity of the posterior soft tissues using MRI or ultrasound, and the variable-magnifying effect of the soft tissue may have resulted in overestimation of the measurements. However, despite these limitations, we believe that our study provides new insight into the risk factors for hip dislocation in patients undergoing primary cementless THA for FNF.

## Conclusion

Hip dislocation in this population may be influenced by a combination of factors, including insufficient stem antetorsion and a lower abduction angle of the acetabular component, especially in patients with deep flexion or internal rotation of the flexed hip joint and knees. This may in turn lead to impingement between the neck of the prosthesis and cup component, resulting in primary impingement and posterior dislocation. Based on our results, a lower abduction angle of the cup combined with insufficient stem antetorsion was a higher risk factors for hip instability and dislocation can be safely deduced. To the best of our knowledge, this is the first time that such a combined risk factors were found for THA, which could remind surgeons to avoid simultaneous occurrence of both in THA surgery. The current study offers new insight into the risk factors for hip dislocation in primary THA for FNF and may aid in reducing the risk of instability and dislocation during THA.

## Data Availability

All data are available upon request from the corresponding author of the present paper.
